# Near-Infrared Optical Constants and Guided-Mode Benchmarking of High-Index MoSe_2_ for Nanophotonics

**DOI:** 10.3390/nano16120747

**Published:** 2026-06-15

**Authors:** Dmitry Yakubovsky, Andrey Vyshnevyy, Dmitriy Grudinin, Bogdan Karpenko, Mikhail Tatmyshevskiy, Timur Kochetkov, Georgy Ermolaev, Aleksey Arsenin, Valentyn Volkov

**Affiliations:** 1 Moscow Center for Advanced Studies, Kulakova Str. 20, Moscow 123592, Russia; vyshnevyy@xpanceo.com (A.V.); igmangym@yandex.ru (B.K.); mihailtatmyshev@inbox.ru (M.T.);; 2 Emerging Technologies Research Center, XPANCEO, Internet City, Emmay Tower, Dubai P.O. Box 393047, United Arab Emirates; grudinin@xpanceo.com (D.G.); ermolaev-georgy@xpanceo.com (G.E.); vsv@xpanceo.com (V.V.)

**Keywords:** molybdenum diselenide, transition metal dichalcogenides, van der Waals materials, anisotropic optical constants, spectroscopic ellipsometry, scanning near-field optical microscopy, integrated photonics, waveguide crosstalk

## Abstract

The integration density of photonic integrated circuits is fundamentally limited by evanescent field overlap and subsequent inter-channel crosstalk. Layered transition metal dichalcogenides (TMDCs) bypass these confinement constraints through intrinsic optical birefringence and high refractive indices. Here, we report the near-infrared optical constants and waveguide dispersion of molybdenum diselenide (MoSe_2_). Ellipsometry performed on centimeter-scale crystals yields an in-plane refractive index of 4.1–4.7 over 1000–2000 nm, with an extinction coefficient close to the sensitivity limit of the fit away from strong excitonic resonances. To validate the anisotropic dielectric tensor at the device scale, scattering-type scanning near-field optical microscopy (s-SNOM) was utilized to map the propagation of transverse-magnetic modes in 235 nm thick exfoliated flakes. Spatial Fourier analysis of the edge-scattered near-field interference yields effective mode indices that precisely match the modeled dispersion. Using the verified dielectric tensor, finite-element simulations demonstrate that single-mode MoSe_2_ waveguides optically outperform equivalent tungsten disulfide (WS_2_) benchmarks. The enhanced evanescent field suppression in the claddings of MoSe_2_ waveguide increases the coupling length by a factor of 3.5, reducing the required routing pitch and enabling a 12.5% direct increase in on-chip integration density. The results identify MoSe_2_ as a high-index anisotropic platform for compact waveguiding in the near-infrared.

## 1. Introduction

Dense photonic integrated circuits require tight optical confinement to minimize individual device footprints [1,2]. In conventional isotropic platforms, such as silicon and silicon nitride, physical scaling is bottlenecked by evanescent field leakage, which causes severe optical crosstalk at sub-wavelength routing pitches [3]. Layered van der Waals (vdW) materials with a strong in-plane covalent bonding and weak out-of-plane vdW forces offer a structural solution to this confinement limit [4]. VdW materials have become a central material class in modern nanophotonics because they combine strong light–matter interaction with crystallographically defined optical anisotropy and broad compatibility with heterogeneous integration [2,3,5,6,7,8]. Within this family, transition metal dichalcogenides (TMDCs) are especially relevant for guided-wave and resonant nanophotonics because bulk and multilayer crystals can simultaneously provide a high refractive index, giant uniaxial optical birefringence, and low loss over selected spectral windows [4,9,10,11,12,13,14]. From an integrated photonics perspective, these properties are not only of fundamental interest. The refractive-index contrast and the anisotropic dielectric response directly determine modal confinement, single-mode design limitations, evanescent field penetration, and the strength of coupling between adjacent optical channels [5,9,15,16]. As a result, reliable optical constants in the near-infrared around the telecommunication band are crucial for the realistic benchmarking of TMDC waveguides, couplers, and other building blocks of photonic integrated circuits.

Molybdenum diselenide (MoSe_2_) is a particularly interesting material of the high-index TMDC class [5]. Prior studies have established that MoSe_2_ can sustain guided exciton–polariton transport in crystal flake waveguides closer to the visible and excitonic spectral range, and highly confined optical modes and high nonlinear response in epitaxially grown MoSe_2_ in the near-infrared range [12,17,18]. The combination of a high in-plane refractive index (*n*_ab_ > 4), low absorption, and giant optical anisotropy, birefringence Δ*n* = *n*_ab_ − *n*_c_ ~ 1, in crystal bulk MoSe_2_ enables the localization of the electromagnetic field at subwavelength scales and provides a single-mode operation in compact photonic waveguides [4,5,9]. In this regard, the design of low-loss near-infrared photonic components requires a device-oriented description that links anisotropic optical constants, experimentally observed guided modes, and engineering figures of merit such as coupling length. However, determination of the dielectric permittivity of vdW crystals is non-trivial, since the studied samples—microcrystals, i.e., flakes—obtained by mechanical exfoliation from the original crystal, often have dimensions of up to several tens of micrometers [19,20]. This restricts the utilization of traditional methods such as spectroscopic ellipsometry and reflectometry for determining the optical constants over a wide spectral range. Moreover, these far-field optical methods have low sensitivity in determining the out-of-plane component of refractive index (*n*_c_) [10]. In contrast, scattering-type scanning near-field optical microscopy (s-SNOM) enables local measurement of the optical response, excitation and propagation of guided modes in vdW materials with spatial resolution exceeding the diffraction limit, and allows verification of the numerical values of the anisotropy of optical constants obtained independently, using spectroscopic ellipsometry [9,10,16,20,21,22].

In this work, we present a detailed characterization of the optical constants and waveguide properties of the high-index crystal MoSe_2_ in the near-infrared by combining the macroscopic spectroscopic ellipsometry, s-SNOM, and electromagnetic modeling to build and test an anisotropic optical model demonstrating the applications of MoSe_2_ in nanophotonics. First, we extract the in-plane optical constants of a macroscopic crystal in the 1000–2000 nm range. Next, we visualize guided TM modes in an exfoliated MoSe_2_ flake and compare the measured modal dispersion with transfer-matrix calculations. Finally, we use the experimentally supported model to benchmark ridge MoSe_2_ waveguides against WS_2_ counterparts in the single-mode regime.

## 2. Materials and Methods

### 2.1. Material Preparation, Morphological and Elemental Characterization

A commercially available ultra-flat large-area bulk MoSe_2_ crystal grown by chemical vapor transport (CVT, 2D Semiconductors, Inc., Phoenix, AZ, USA) was used for ellipsometric characterization. The crystal composition was examined by energy-dispersive X-ray spectroscopy (EDS, Bruker QUANTAX EDX, Bruker Corporation, Billerica, MA, USA) in a scanning electron microscope setup (SEM, JEOL JSM-7001F, JEOL Ltd., Tokyo, Japan) working in secondary electron imaging mode. The value of the acceleration voltage was 15 keV. The EDS spectrum was analyzed using the software provided by Bruker. The crystal phase of MoSe_2_ was assessed by the Raman spectrometer Horiba LabRAM HR Evolution (HORIBA Ltd., Kyoto, Japan) equipped with a confocal microscope with objective ×100/N.A. = 0.90 and 1800 lines/mm diffraction grating, working at an excitation wavelength of 532 nm. For near-field measurements, MoSe_2_ flakes were obtained by mechanical exfoliation from the same parent crystal and transferred onto a SiO_2_(285 nm)/Si substrate. Optical microscopy was used to identify flakes of sufficient lateral size, and atomic force microscopy (AFM, NT-MDT Spectrum Instruments, NT-MDT Ltd., Moscow, Russian Federation) in contact mode was used to determine the flake thickness. The cantilevers employed were NSA01 tips (TipsNano OÜ, Tallinn, Estonia), characterized by a spring constant of 5.1 N/m, a tip radius of less than 10 nm, and a resonant frequency of 150 kHz. Analysis of AFM scans was performed using Gwyddion (ver. 2.71) software.

### 2.2. Spectroscopic Ellipsometry

The optical constants of the macroscopic MoSe_2_ crystal were measured with a variable-angle spectroscopic ellipsometer (V-VASE, J.A. Woollam Co., Linkoln, NE, USA) in the wavelength range 1000–2000 nm. A large-area crystal (~1 cm^2^) was required because the ellipsometer spot size was approximately ~1.5 mm. The ellipsometric parameters *Ψ* and Δ were recorded in reflection geometry at incidence angles of 50°, 55°, and 60° with a wavelength step of 5 nm. The data were fitted in WVASE using a parallel-sided slab model that included backside reflection. The dielectric response was parameterized with the Tauc–Lorentz dispersion model, which preserves the Kramers–Kronig consistency and has been broadly used for TMDC optical constants extraction [5,12]. The out-of-plane component of the refractive index *n*_c_ was determined using the imaging ellipsometer (Accurion EP4) in the nulling mode. Ellipsometry spectra were recorded for MoSe_2_ flake with thickness of 100 nm and in the NIR spectral range. For ellipsometry analysis, the fitting method similar to the procedure described in previous works was used [10].

### 2.3. Scattering-Type Scanning Near-Field Optical Microscopy

Near-field imaging of guided modes was performed with a commercial s-SNOM system (neaSNOM, Neaspec GmbH, Haar, Germany) operated in reflection mode. A tunable continuous-wave fiber-coupled laser (Agilent 81600B) with a range of 1460–1640 nm was used as the excitation source. The optical probe was a metalized AFM tip (NanoWorld, ARROW-NCPt-50) oscillating at *Ω* ≈ 280 kHz. A pseudo-heterodyne interferometric scheme was employed, and the detector signal was demodulated third harmonic to isolate the background-suppressed near-field amplitude and phase [23]. The excitation polarization was chosen to launch TM-polarized guided modes in the flake. To select a suitable planar waveguide thickness, the TM-mode dispersion of the MoSe_2_/SiO_2_/Si stack was first evaluated by the transfer-matrix method [24] for the measured in-plane response and the adopted out-of-plane refractive index.

The near-field fringe pattern was analyzed in the Fourier domain using line profiles extracted perpendicularly to the flake edge. In accordance with the established interpretation of guided-mode s-SNOM in anisotropic TMDCs flakes, the observed pattern is attributed to interference between the incident field and a tip-launched guided mode that is scattered at the flake boundary. The Fourier peak position yields the apparent normalized in-plane momentum *n*_s-SNOM_. The effective mode index was then extracted as $n_{\mathrm{eff}}\;=\;n_{s-\mathrm{SNOM}}\;+\;\cos\alpha \;\cdot \sin\beta$, where *α* is the angle between the illumination wavevector and its projection onto the sample plane, and *β* is the angle between the in-plane projection and the flake edge. For the present geometry, *α* = 45° and *β* = 90°.

### 2.4. Numerical Modeling

Ridge waveguides with MoSe_2_, WS_2_ and Si cores on a SiO_2_ substrate and air cladding were analyzed using the finite-element method in COMSOL Multiphysics. The simulation domain was 8 × 8 μm with PEC boundary. Mesh size varied from 20 nm at the waveguide core to 250 nm near the domain boundary. The crystal optic axis was assumed to be normal to the substrate, consistent with the layered-flake geometry. To quantify coupling between neighboring waveguides, even and odd supermodes of a coupled-waveguide pair were calculated for different center-to-center spacings *d*. The coupling length was then obtained from [25]:

$$L_{c}\;=\;\lambda /\left[2\left|n_{\mathrm{even}}-\;n_{\mathrm{odd}}\right|\right].$$

Coupling length was optimized at core widths and heights, at which waveguide operates in the single-mode regime. Since *L_c_* grows with the increase in core height, the optimal waveguide size is found at the line separating single and double-mode operation regimes.

## 3. Results and Discussion

**Figure 1:**
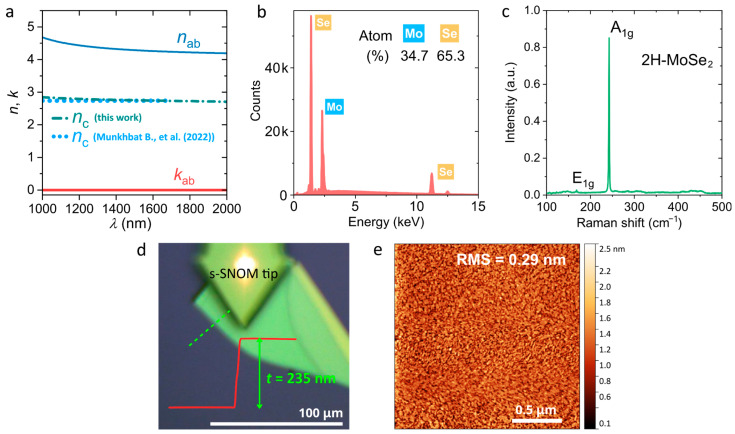
(**a**) Near-infrared optical constants of MoSe_2_. The solid blue curve shows the in-plane refractive index *n*_ab_ extracted by spectroscopic ellipsometry, the red curve shows the in-plane extinction coefficient *k*_ab_, and the dashed green and blue curve shows the out-of-plane refractive index *n*_c_ determined independently and taken from the literature [12]. (**b**) EDS spectrum of the parent crystal confirming near-stoichiometric composition. (**c**) Raman spectrum of MoSe_2_ exfoliated flake. (**d**) Optical micrograph of the exfoliated MoSe_2_ flake used for s-SNOM measurements. The red trace and green arrow indicate the AFM step profile yielding *t* = 235 nm. The scale bar is 100 μm. (**e**) AFM scan of the MoSe_2_ flake surface with RMS roughness value over 2 × 2 μm^2^.

For the initial determination of the in-plane optical constants, spectroscopic ellipsometry was applied to the molybdenum diselenide (MoSe_2_) crystal. Measurements were performed using a Woollam V-VASE ellipsometer in the wavelength range *λ* = 1000–2000 nm, which is important for telecommunications applications. A large-area crystal (~1 cm^2^), synthesized by CVT, was chosen as the MoSe_2_ sample. In the experiment, the choice of a macroscopic crystal was dictated by the minimum spot size of the ellipsometer measurement beam, which is approximately ~1.5 mm in diameter. Measurements of the ellipsometric parameters *Ψ* and Δ were performed in reflection mode (see Methods). To determine the in-plane refractive index *n*_ab_ and extinction coefficient *k*_ab_ of the MoSe_2_ crystal, a layered model including backside reflection was used. For the dielectric function, the Tauc–Lorentz oscillator dispersion model was employed in fitting the *Ψ* and Δ spectra, providing analytical satisfaction of the Kramers–Kronig relations, similarly to approaches used for other TMDCs, using the WVASE software [9,10]. The results of the ellipsometry data analysis for MoSe_2_ are presented in Figure 1a. The in-plane refractive index *n*_ab_ varies smoothly in the range of 4.1–4.7, remaining well above the values typical of conventional dielectric photonic materials in the near-infrared [5,10]. At the same time, the fitted extinction coefficient *k*_ab_ in the near-infrared window is below the sensitivity of the present ellipsometric fit, which is consistent with previous studies [12,18]. The performed ellipsometry method with a large-area MoSe_2_ crystal is only effective for determining in-plane optical constants, but not the out-of-plane component *n*_c_, due to the high uncertainty in the sample thickness. Thus, to determine the out-of-plane refractive index *n*_c_ with sufficient accuracy to take into account the anisotropy in further MoSe_2_ waveguides studies, the ellipsometry of the separate flake with a certain thickness was employed (see Methods), yielding the results in Figure 1a within ~0.1 agree with *n*_c_ curve from reference [12].

The s-SNOM technique allows for the independent verification of the optical constants of vdW crystals and confirmation of the magnitude of optical anisotropy; s-SNOM was used to measure the propagation of photonic modes in MoSe_2_ [10]. To this end, planar waveguides were fabricated by mechanically exfoliating MoSe_2_ flakes onto a SiO_2_(285 nm)/Si substrate from a macroscopic crystal. Suitable MoSe_2_ flakes with sufficient area and the required thickness for the propagation of the fundamental photonic TM mode in the wavelength range near 1500 nm were identified using optical microscopy and AFM. The range of thicknesses required for the propagation of a single TM mode was determined by numerical calculation using the transfer-matrix method [24] and the optical constants of MoSe_2_ in Figure 1a, yielding thickness values from 200 to 370 nm. This choice is important because it enables a direct comparison between measured and calculated modal dispersion without the ambiguity introduced by higher-order guided states. The AFM step profile on a chosen flake yielded a thickness of 235 ± 5 nm (see inset in Figure 1c), which falls inside the calculated single-mode interval for the planar TM mode near *λ* = 1.5 μm and was selected for s-SNOM measurements. The roughness of 0.29 nm (Figure 1e) confirms an atomically flat top surface which would result in reduced defect-related losses in waveguiding applications. In addition, the EDS spectrum in Figure 1b confirms the expected stoichiometry of the parent crystal, with measured atomic fractions of 34.7% for Mo and 65.3% for Se, corresponding closely to the 1:2 composition of MoSe_2_. To confirm the crystal phase, we applied Raman spectroscopy measurements of MoSe_2_ flake shown in Figure 1c, which correspond to bulk 2H-MoSe_2_ and are characterized by two vibrational modes: the in-plane *E*_1g_ at ~168 cm^−1^ and the out-of-plane *A*_1g_ peak at ~242 cm^−1^ [26]. Bulk 2H-MoSe_2_ has an indirect band gap of ~1.2 eV [5,12]. The photon energies (0.76–0.84 eV) considered in the work for studying the propagation of photonic modes in 2H-MoSe_2_ are below the fundamental band gap, which supports the use of a low-absorption NIR dielectric window.

**Figure 2:**
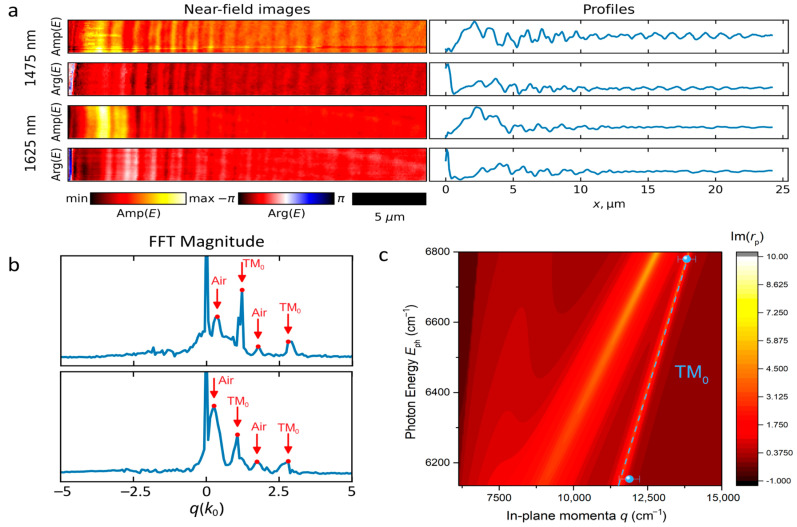
Near-field characterization of guided modes in MoSe_2_ planar waveguide. (**a**) s-SNOM amplitude and phase images acquired near the flake edge at excitation wavelengths of 1475 and 1625 nm, together with representative line profiles. (**b**) Amplitude of the real part of the complex Fourier transform spectrum of the signals from (**a**) at 1475 nm and 1625 nm (frequency normalized to the effective index *q*/*k*_0_ = n_s-SNOM_). (**c**) Experimental dispersion points of the planar TM_0_ mode (blue dots) compared with transfer-matrix calculations (solid orange line) based on the anisotropic optical constants from Figure 1a.

Next, to study photonic modes in MoSe_2_ planar waveguides, the s-SNOM metallic tip is illuminated with laser radiation in the 1460–1640 nm range in TM polarization (see Section 2.3 for more details). Scanning the tip perpendicular to the crystal edge allows mapping of interference fringes. The resulting s-SNOM amplitude and phase images recorded near the flake edge at wavelengths of 1475 and 1625 nm are shown in Figure 2a. Both wavelengths reveal a clear sequence of near-field fringes extending inward from the flake boundary, which is consistent with the excitation of a guided TM_0_ mode in the planar MoSe_2_ waveguide. The fringe periodicity is directly related to the mode wavelength (*λ*_p_) and, consequently, to the effective mode index (*n*_eff_ = *λ*_0_/*λ*_p_, where *λ*_0_ is the free-space wavelength). The effective refractive index of the propagating TM mode in an anisotropic planar MoSe_2_ waveguide depends on both the in-plane (*n*_ab_) and out-of-plane (*n*_c_) refractive indices. Thus, by determining *n*_eff_ and comparing it with theoretical calculations of the dispersion curve using the transfer-matrix method, it is possible to verify the anisotropic optical constants. To determine *n*_eff_, complex fast Fourier transform of line profiles extracted from the near-field images was performed and the corresponding Fourier spectra are shown in Figure 2b. The dominant peaks (at *q*(*k*_0_) ~ 1.1) assigned to the TM_0_ mode were used to determine the in-plane modal momenta, while additional peaks in the Fourier spectra arise due to the propagation of the wave in the air and do not affect the position of the peak corresponding to the desired TM_0_ mode. After applying the geometric frequency shift described in Section 2.3, the extracted effective indices at 1475 and 1625 nm were compared with transfer-matrix calculations of the energy (*E* ~ 1/*λ*_0_)-momentum (*q* = 1/*λ*_p_) dispersion relation of the waveguide mode for the anisotropic MoSe_2_/SiO_2_/Si stack. As shown in Figure 2c, the experimental effective indices’ points follow the calculated dispersion curve. This agreement does not by itself constitute a full independent reconstruction of the complete dielectric tensor, because the out-of-plane response was measured from an exfoliated MoSe_2_ flake, but it does provide an important consistency check: the measured in-plane optical constants *n*_ab_, *k*_ab_, *n*_c_, and the flake thickness together describe the observed guided-mode dispersion without the need for further adjustable parameters. Considering that the uncertainty in the determination of the *n*_eff_ of guided modes is about 0.05 (Figure 2c), we find that the agreement between s-SNOM measurement results and transfer-matrix calculations means that the obtained *n*_c_ values’ error is below 0.08 for wavelengths of 1475 and 1625 nm. Therefore, both the literature out-of-plane index *n*_c_ values from reference [12] and our new values directly describe the observed guided-mode dispersion in the telecom band without the need for further adjustable parameters.

**Figure 3:**
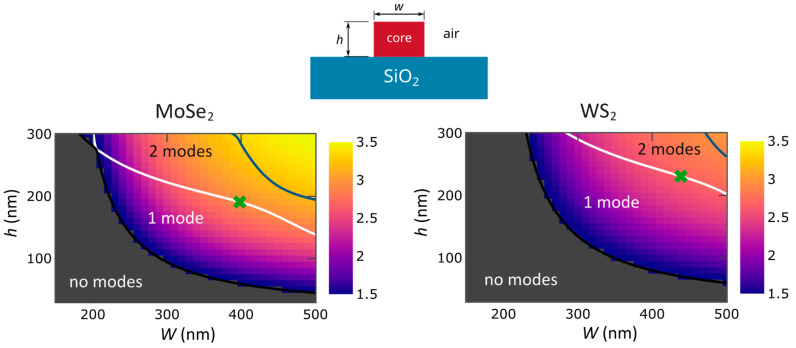
Effective-index maps for the fundamental waveguide mode of rectangular waveguides with MoSe_2_ and WS_2_ cores on a SiO_2_ substrate as a function of core width and height. The black, white, and blue lines separate the region where no modes are supported from the regions where one, two, or more modes are supported, respectively. The green cross marks the parameters that provide minimal crosstalk between waveguides whose core centers are separated by a distance *d* = 1 μm.

Using the experimentally supported optical model, we next evaluated the prospects of using MoSe_2_ in nanophotonics by numerical modeling of the behavior of waveguides based on this material. Characteristics of MoSe_2_ waveguides were compared with those of WS_2_—another TMDC with a high refractive index and optical anisotropy—whose waveguiding performance has previously been shown to be better than silicon, the backbone material of modern nanophotonics [9]. First, the effective indices of the supported waveguide modes were calculated (see Methods). This allowed determination of the number of supported waveguide modes as a function of the waveguide core width (*w*) and height (*h*), as well as the construction of effective-index maps for the fundamental mode, i.e., the mode with the highest index. From the nanophotonic viewpoint, the effective index is an important parameter characterizing the degree of field confinement within the waveguide core. In particular, it determines the size of resonators and modulators, and also governs radiation losses in bent waveguide sections. Furthermore, the higher the effective index, the faster the evanescent field decays in the waveguide cladding, leading to reduced crosstalk between adjacent waveguides and, consequently, enabling denser integration of waveguides on a chip, thereby enhancing the performance of photonic integrated circuits [9]. In Figure 3, the comparison of the effective-index maps for MoSe_2_ and WS_2_ rectangular waveguides shows that the MoSe_2_ core supports modes with a higher effective index at smaller core dimensions, which is expected due to the higher refractive index of MoSe_2_. On the other hand, for equal dimensions, MoSe_2_ waveguides support more modes, whereas in practice, a single-mode regime is important, as it allows better control of signal transmission through the waveguide. Nevertheless, in the single-mode regime, i.e., at smaller core dimensions, MoSe_2_ waveguides exhibit a higher effective index than WS_2_ waveguides, suggesting weaker crosstalk compared to WS_2_.

To test this hypothesis, the coupling length *L*_c_, which characterizes the mutual influence of adjacent waveguides, was calculated. After propagating this distance along the waveguide, the mode completely transfers from one waveguide to the neighboring one (see Methods for the details on crosstalk calculations). The calculation results show that for a fixed waveguide core width *w*, *L*_c_ increases with core height; therefore, the optimal core dimensions should lie on the cutoff curve of the second waveguide mode (the white curve in Figure 3). For a fixed center-to-center distance *d* = 1 μm between waveguides, the maximum achievable coupling length *L*_c_ for MoSe_2_ waveguides exceeds 15 mm, which is approximately 3.5 times higher than the same value for WS_2_ waveguides, which is 4.3 mm. To provide more context, the maximum *L_c_* for similar silicon waveguides is just 0.59 mm, which is almost an order of magnitude below the same value for WS_2_.

**Figure 4:**
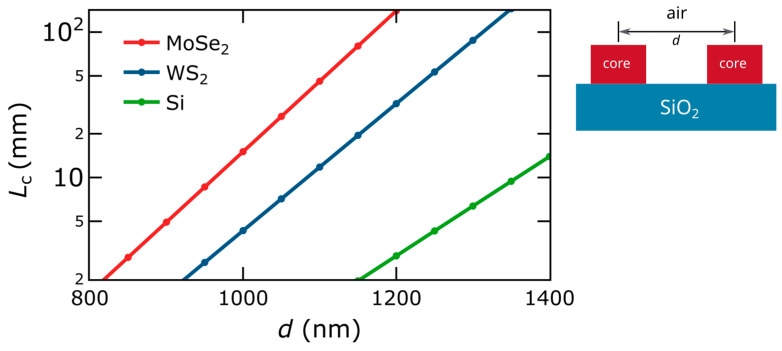
Coupling length *L*_c_ of the fundamental mode as a function of the center-to-center distance *d* between two parallel waveguide cores. For each value of *d*, the core dimensions were optimized within the single-mode regime. Beside the plot is the coupled-waveguide geometry used in the calculations.

A more detailed investigation of the dependence of *L*_c_ on *d* (Figure 4) shows that the advantage of MoSe_2_ over WS_2_ and over Si increases as the distance between waveguides grows. At the same time, if we compare the minimum distance between waveguides at which *L*_c_ = 10 mm, which is sufficient for constructing global interconnects on a chip, then MoSe_2_ (*d*_min_ = 960 nm) outperforms WS_2_ (*d*_min_ = 1080 nm) by 12.5% and silicon by (*d*_min_ = 1350 nm) by 40%. This enhancement is very significant and hard to achieve considering the near-exponential dependence of the coupling length on the distance between waveguides, which is caused by the exponential decay of the waveguide mode field outside the waveguide core. Moreover, there exist methods for further increasing integration density by engineering the space between waveguides, which can not only reduce the distance between waveguides but also almost completely suppress waveguide coupling [25,27].

Looking forward, the availability of scalable, high-index MoSe_2_ structures provides a material platform for realizing all vdW microcavity photonics, high-Q resonant photonics and the integration of heterogeneous photonic circuits, etc. [28,29]. Such scalability has remained hard to achieve until now, as previous demonstrations of TMDC-based structures have predominantly relied on exfoliated flakes, whose lateral dimensions are limited to tens or hundreds of micrometers. For practical mm-cm photonic routing, MoSe_2_ waveguides would require larger-area crystals or wafer-scale grown TMDC films with controlled thickness and roughness. However, recent advances in inch-scale MoSe_2_-layer growth via molecular beam epitaxy (MBE) and subsequent patterning using electron lithography ensure a level of scalability that is crucial for the industrial fabrication of nanophotonic structures [18]. Given that grown 3D MoSe_2_ layers exhibit optical properties comparable to those of exfoliated crystals, our results, aimed at clarifying the anisotropic optical properties, assessing the integration density of MoSe_2_ based waveguides in comparison with other TMDCs and providing the design basis for such devices, establish MoSe_2_ as a promising platform for integrated nanophotonics in the telecommunication range.

## 4. Conclusions

We have evaluated bulk-like MoSe_2_ as a near-infrared waveguiding material by combining spectroscopic ellipsometry, s-SNOM near-field imaging, and electromagnetic modeling. The fitted in-plane refractive index remains between 4.1 and 4.7 from 1000 to 2000 nm, while the fitted extinction coefficient is close to zero within the sensitivity of the fit. Guided TM_0_ modes imaged at 1475 and 1625 nm yield effective indices consistent with transfer-matrix calculations based on the measured in-plane response, the measured flake thickness, and an out-of-plane index. This agreement supports the internal consistency of the anisotropic optical model. Waveguide benchmarking further showed that, in the single-mode regime, MoSe_2_ provides higher effective indices and longer coupling lengths than another high-refractive-index anisotropic material, WS_2_. This leads to a reduction in crosstalk by approximately a factor of 3.5, demonstrating that MoSe_2_ waveguides are more compact and effective, which can be used to increase the integration density and reliability of photonic circuits. These results support MoSe_2_ as a high-index anisotropic material platform for compact near-infrared photonic routing.

## Data Availability

The original contributions presented in this study are included in the article. Further inquiries can be directed to the corresponding authors.

## References

[1] Bogaerts W., Pérez D., Capmany J., Miller D.A.B., Poon J., Englund D., Morichetti F., Melloni A. (2020). Programmable Photonic Circuits. Nature.

[2] de Abajo F.J.G., Basov D.N., Koppens F.H.L., Orsini L., Ceccanti M., Castilla S., Cavicchi L., Polini M., Gonçalves P.A.D., Costa A.T. (2025). Roadmap for Photonics with 2D Materials. ACS Photonics.

[3] Song S., Rahaman M., Jariwala D. (2024). Can 2D Semiconductors Be Game-Changers for Nanoelectronics and Photonics?. ACS Nano.

[4] Ling H., Li R., Davoyan A.R. (2021). All van Der Waals Integrated Nanophotonics with Bulk Transition Metal Dichalcogenides. ACS Photonics.

[5] Zotev P.G., Wang Y., Andres-Penares D., Severs-Millard T., Randerson S., Hu X., Sortino L., Louca C., Brotons-Gisbert M., Huq T. (2023). Van Der Waals Materials for Applications in Nanophotonics. Laser Photon. Rev..

[6] Feng Y., Chen R., He J., Qi L., Zhang Y., Sun T., Zhu X., Liu W., Ma W., Shen W. (2023). Visible to Mid-Infrared Giant in-Plane Optical Anisotropy in Ternary van Der Waals Crystals. Nat. Commun..

[7] Ma G., Shen W., Sanchez D.S., Yu Y., Wang H., Sun L., Wang X., Hu C. (2023). Excitons Enabled Topological Phase Singularity in a Single Atomic Layer. ACS Nano.

[8] Grudinin D.V., Ermolaev G.A., Baranov D.G., Toksumakov A.N., Voronin K.V., Slavich A.S., Vyshnevyy A.A., Mazitov A.B., Kruglov I.A., Ghazaryan D.A. (2023). Hexagonal Boron Nitride Nanophotonics: A Record-Breaking Material for the Ultraviolet and Visible Spectral Ranges. Mater. Horiz..

[9] Vyshnevyy A.A., Ermolaev G.A., Grudinin D.V., Voronin K.V., Kharichkin I., Mazitov A., Kruglov I.A., Yakubovsky D.I., Mishra P., Kirtaev R.V. (2023). Van Der Waals Materials for Overcoming Fundamental Limitations in Photonic Integrated Circuitry. Nano Lett..

[10] Ermolaev G.A., Grudinin D.V., Stebunov Y.V., Voronin K.V., Kravets V.G., Duan J., Mazitov A.B., Tselikov G.I., Bylinkin A., Yakubovsky D.I. (2021). Giant Optical Anisotropy in Transition Metal Dichalcogenides for next-Generation Photonics. Nat. Commun..

[11] Khurgin J.B. (2022). Expanding the Photonic Palette: Exploring High Index Materials. ACS Photonics.

[12] Munkhbat B., Wróbel P., Antosiewicz T.J., Shegai T.O. (2022). Optical Constants of Several Multilayer Transition Metal Dichalcogenides Measured by Spectroscopic Ellipsometry in the 300–1700 Nm Range: High Index, Anisotropy, and Hyperbolicity. ACS Photonics.

[13] Zograf G., Polyakov A.Y., Bancerek M., Antosiewicz T.J., Küçüköz B., Shegai T.O. (2024). Combining Ultrahigh Index with Exceptional Nonlinearity in Resonant Transition Metal Dichalcogenide Nanodisks. Nat. Photonics.

[14] Nørgaard M., Yezekyan T., Rolfs S., Frydendahl C., Mortensen N.A., Zenin V.A. (2025). Near-Field Refractometry of van Der Waals Crystals. Nanophotonics.

[15] Zotev P.G., Bouteyre P., Wang Y., Randerson S.A., Hu X., Sortino L., Wang Y., Shegai T., Gong S.-H., Tittl A. (2025). Nanophotonics with Multilayer van Der Waals Materials. Nat. Photonics.

[16] Hu D., Yang X., Li C., Liu R., Yao Z., Hu H., Corder S.N.G., Chen J., Sun Z., Liu M. (2017). Probing Optical Anisotropy of Nanometer-Thin van Der Waals Microcrystals by near-Field Imaging. Nat. Commun..

[17] Hu F., Luan Y., Scott M.E., Yan J., Mandrus D.G., Xu X., Fei Z. (2017). Imaging Exciton–polariton Transport in MoSe_2_ Waveguides. Nat. Photonics.

[18] Pruszyńska-Karbownik E., Fąs T., Brańko K., Yavorskiy D., Stonio B., Bożek R., Karbownik P., Wróbel J., Czyszanowski T., Stefaniuk T. (2026). Optical Bound States in the Continuum in Subwavelength Gratings Made of an Epitaxial van Der Waals Material. ACS Nano.

[19] Li Y., Kuang G., Jiao Z., Yao L., Duan R. (2022). Recent Progress on the Mechanical Exfoliation of 2D Transition Metal Dichalcogenides. Mater. Res. Express.

[20] Yakubovsky D.I., Grudinin D.V., Pak N.V., Leiman V.G., Arsenin A.V. (2025). Scanning near-Field Optical Microscopy Characterization of WSe_2_ and MoSe_2_ Planar Waveguides. Bull. Russ. Acad. Sci. Phys..

[21] Basov D.N., Fogler M.M., García de Abajo F.J. (2016). Polaritons in van Der Waals Materials. Science.

[22] Fei Z., Rodin A.S., Gannett W., Dai S., Regan W., Wagner M., Liu M.K., McLeod A.S., Dominguez G., Thiemens M. (2013). Electronic and Plasmonic Phenomena at Graphene Grain Boundaries. Nat. Nanotechnol..

[23] Ocelic N., Huber A., Hillenbrand R. (2006). Pseudoheterodyne Detection for Background-Free near-Field Spectroscopy. Appl. Phys. Lett..

[24] Passler N.C., Paarmann A. (2017). Generalized 4 × 4 Matrix Formalism for Light Propagation in Anisotropic Stratified Media: Study of Surface Phonon Polaritons in Polar Dielectric Heterostructures. J. Opt. Soc. Am. B.

[25] Grudinin D., Matveeva O., Ermolaev G., Vyshnevyy A., Arsenin A., Volkov V. (2023). Reduction in Crosstalk between Integrated Anisotropic Optical Waveguides. Photonics.

[26] Wu C.T., Hu S.Y., Tiong K.K., Lee Y.C. (2017). Anisotropic effects in the Raman scattering of Re-doped 2H-MoSe_2_ layered semiconductors. Results Phys..

[27] Mia M.B., Ahmed S.Z., Ahmed I., Lee Y.J., Qi M., Kim S. (2020). Exceptional Coupling in Photonic Anisotropic Metamaterials for Extremely Low Waveguide Crosstalk. Optica.

[28] Wang Z.Y., Cui X., Liapis A.C., Shao H.-R., Cheng X., Yang J., Shang N., Zhang W., Kaaripuro H., Muñoz J.C.A. (2026). All-van der Waals microcavities for low-loss nonlinear photonics. Nat. Mater..

[29] Meng Y., Feng J., Han S., Xu Z., Mao W., Zhang T., Kim J.S., Roh I., Zhao Y., Kim D.-H. (2023). Photonic van der Waals integration from 2D materials to 3D nanomembranes. Nat. Rev. Mater..

